# Regional Gene Expression Patterns are Associated with Functional Connectivity Alterations in Major Depressive Disorder with Anxiety Symptoms

**DOI:** 10.31083/AP39865

**Published:** 2025-04-21

**Authors:** Chengfeng Chen, Wuyou Bao, Runhua Wang, Wen Qin, Bin Zhang, on behalf of REST-meta-MDD Consortium

**Affiliations:** ^1^Department of Psychiatry, The Aﬃliated Brain Hospital of Guangzhou Medical University, 510370 Guangzhou, Guangdong, China; ^2^Department of Psychiatry, Guangzhou Medical University, 511436 Guangzhou, Guangdong, China; ^3^Institute of Mental Health, Tianjin Anding Hospital, Tianjin Medical University, 300222 Tianjin, China; ^4^Department of Radiology and Tianjin Key Laboratory of Functional Imaging, Tianjin Medical University General Hospital, 300070 Tianjin, China; ^5^Mental Health Center of Tianjin University, Tianjin Anding Hospital, 300210 Tianjin, China

**Keywords:** major depressive disorder, anxiety, functional connectivity, gene expression

## Abstract

**Background:**

Understanding gene expression and functional connectivity (FC) changes in depressed patients with anxiety can help develop personalized therapies. Herein we examine the link between transcriptome data and FC differences in patients with major depressive disorder with significant anxiety (MDD/ANX+) and patients with major depressive disorder without significant anxiety (MDD/ANX-).

**Methods:**

We compared the FC between the MDD/ANX+ group (n = 294) and the MDD/ANX- group (n = 218) to identify FC differences at both edge-based and network levels. Using the Allen Human Brain Atlas, we performed partial least squares regression analysis to identify genes associated with the observed FC disparities, followed by a functional enrichment analysis.

**Results:**

The results from both edge-based and network-level FC analyses consistently indicated significantly increased FC between the subcortical network (SC) and visual network, as well as between the SC and dorsal attention network, in the MDD/ANX+ group compared with the MDD/ANX- group. Additionally, transcriptome-neuroimaging correlation analysis revealed that the expression of 1066 genes was spatially correlated with the FC differences between the MDD/ANX+ and MDD/ANX- groups. These genes were enriched in translation at synapses and adenosine triphosphate (ATP) generation.

**Conclusions:**

Our results indicate that gene expression variations in synaptic translation and ATP generation may affect FC and anxiety risk in MDD patients.

## Main Points

1. To our knowledge, this is the first study to investigate gene expression related to FC differences between patients with MDD/ANX+ and patients with MDD/ANX-.

2. Increased FC was observed between the SC and visual network (VN), and between the SC and dorsal attention network (DAN), in the MDD/ANX+ group.

3. Certain genes related to synaptic translation and ATP generation may modulate the FC differences between the two groups.

## 1. Introduction

Patients with major depressive disorder (MDD) frequently exhibit anxiety symptoms, with nearly two-thirds of MDD patients experiencing clinical anxiety [1]. Those with comorbid anxiety symptoms tend to show greater disease severity, an earlier onset, increased suicidal tendencies, prolonged duration of illness, comorbid substance abuse, and heightened resistance to existing therapeutic interventions [2]. This highlights the need for new treatments that target and personalize treatment for MDD with anxiety.

Advancing the understanding of the underlying neuroimaging basis of MDD with anxiety could offer the potential to identify new personalized neuromodulatory targets and thereby improve treatments [2, 3]. There have been some studies exploring the neurobiological mechanisms underlying MDD with significant anxiety symptoms. A study using machine learning to predict MDD in patients with significant anxiety, based on multimodal neuroimaging, found that MDD patients with significant anxiety demonstrated increased functional connectivity (FC) between the left middle temporal gyrus and both the left medial superior frontal gyrus and the temporal pole, compared with MDD patients without significant anxiety [3]. Additionally, some studies have found that decreased FC between the amygdala and the executive control network (ECN) [4] and the default mode network (DMN) [5] was associated with greater anxiety in MDD. These results are heterogeneous, which may be attributed to the relatively small sample sizes in these studies.

The mechanisms underlying FC effects in MDD patients with significant anxiety remain unclear. Recent studies support the hypothesis that regional differences in gene expression may influence FC [6, 7]. Genome-wide gene expression levels have been measured in post-mortem brain tissues and are publicly accessible through resources including the Allen Human Brain Atlas (AHBA) [8]. This availability has facilitated the integration of functional magnetic resonance imaging (fMRI) data and AHBA gene expression profiles, propelling advances in neuroimaging transcriptomics. It allows for the identification of genes whose expression patterns reflect anatomical changes, thereby linking molecular functions to brain organization. Moreover, numerous studies have aimed to elucidate the genetic foundations of imaging abnormalities in psychiatric disorders by analyzing spatial correlations between neuroimaging phenotypes and brain gene expression [6, 9, 10]

**Fig. 1.**
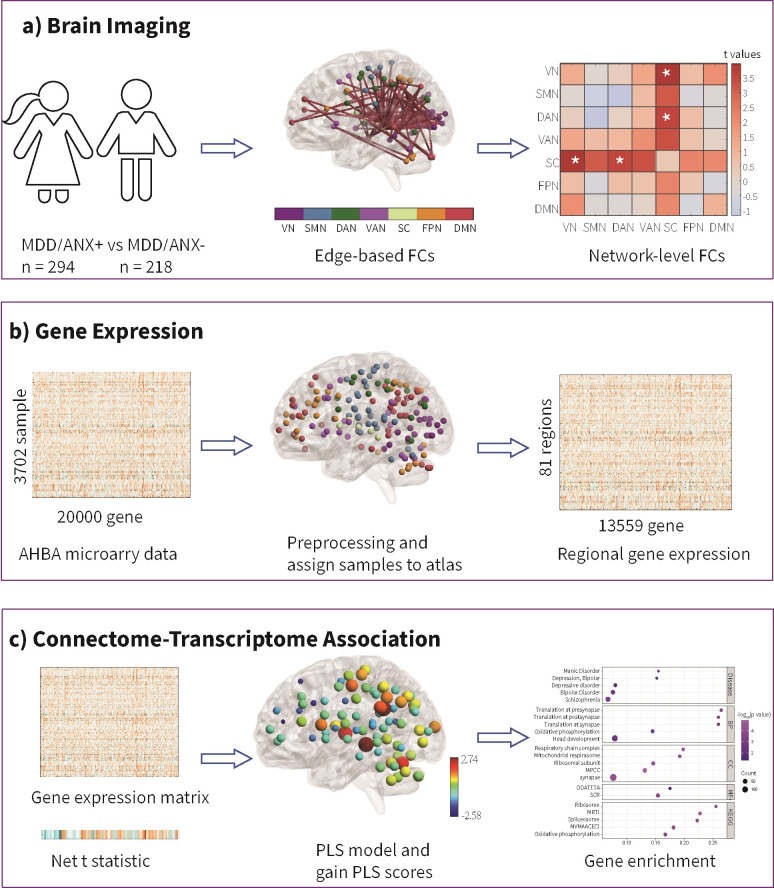
**Schematic overview of the study design and analysis pipeline**. (a) Brain imaging analysis: comparisons of edge-based and network-level functional connectivities (FCs) between the MDD/ANX+ and MDD/ANX- groups were conducted. These comparisons used a two-sample *t*-test and were corrected using the network-based statistics (NBS) method within DPABI 8.1. (b) Gene expression data processing: raw gene expression microarray data were obtained from the AHBA and further processed using the abagen toolbox for transcriptome data. After primary processing steps, including tissue sample mapping and sample assignment, a regional gene expression matrix for the Dosenbach atlas was obtained, and only tissue samples from the left hemisphere were included, as all six donors had expression data in the left hemisphere, while only two donors had samples in the right hemisphere. (c) Connectome-transcriptome association analysis: a partial least squares (PLS) regression model was constructed to investigate the spatial correlation between differences in FCs between MDD/ANX+ and MDD/ANX- and the gene expression matrix. The net t-value was calculated as the sum of positive t-values minus the sum of absolute negative t-values for each region, representing the FC differences between MDD/ANX+ and MDD/ANX-. Genes exhibiting PLS weights exceeding a |z|-score of 3 were selected and submitted to ToppGene for enrichment analyses. Note: MDD, major depressive disorder; MDD/ANX+, MDD patients with significant anxiety; MDD/ANX-, MDD patients without significant anxiety; FCs, functional connectivities; AHBA, Allen Human Brain Atlas; PLS, partial least squares. * indicates significance.

We hypothesize that variations in specific gene expression could influence FC in patients with MDD, potentially conferring a risk for anxiety in MDD. Using fMRI data from 512 individuals with MDD, we compared differences in FC between MDD patients with and without significant anxiety, both at the edge-based and network levels. Subsequently, using the AHBA, we performed spatial correlation analyses of neuroimaging and transcriptomic data to identify genes associated with FC differences in MDD with anxiety. Follow-up analyses included functional enrichment and insights into the functions of the identified genes. An overview of the study design and analysis pipeline is shown in Fig. 1.

## 2. Methods

### 2.1 Participants

**Fig. 2.**
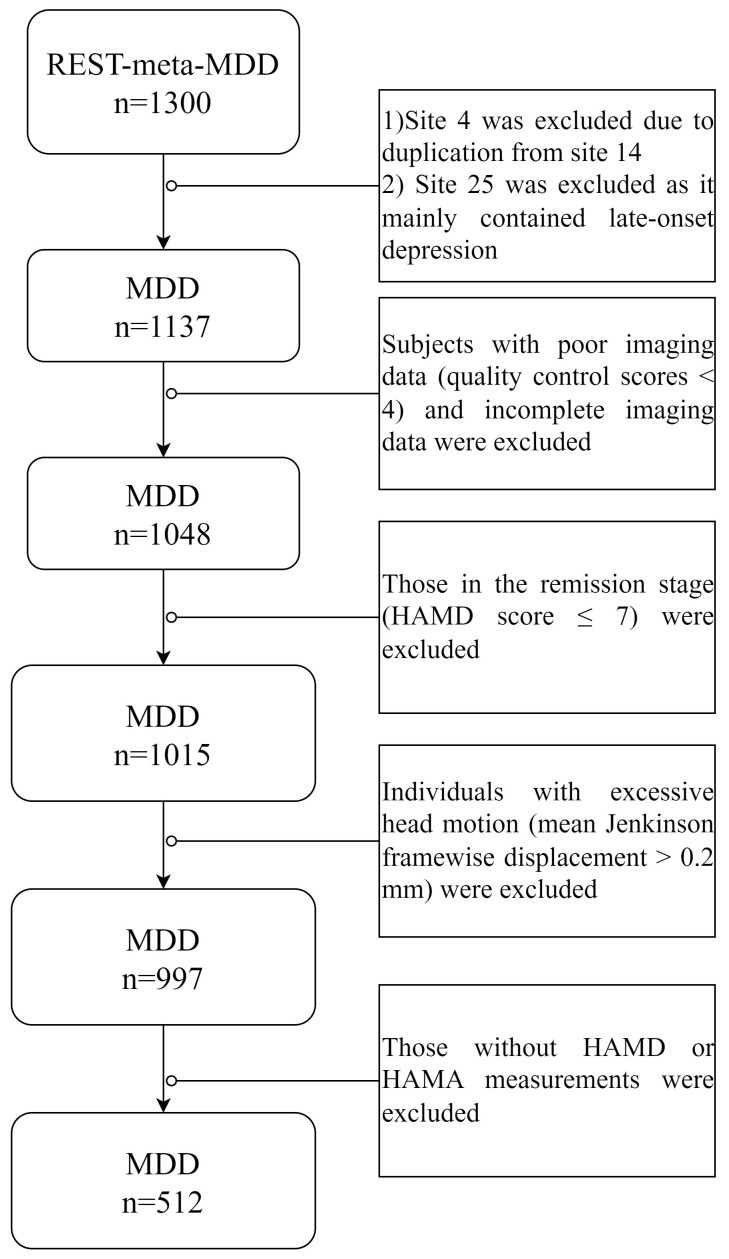
**Selection of individuals from the REST-meta-MDD data**. Note. MDD, MDD patients; HAMD, 17-item Hamilton Depression Rating Scale; HAMA, Hamilton Anxiety Rating Scale.

A total of 512 individuals were chosen from the REST-meta-MDD consortium’s 1300 MDD patient dataset (http://rfmri.org/REST-meta-MDD) for this study [11]. The included participants were from nine sites, with site sample sizes ranging from 6 to 240 individuals each. Details of the sample sizes for each study site are provided in **Supplementary Table 1**. The exclusion criteria were: (1) sites 4 and 25 were excluded because they either duplicated data from site 14 or predominantly included elderly depression cases (primarily aged over 50 years) and patients in remission; (2) subjects with substandard imaging data (quality control scores <4, as assessed by visual inspection) or with excessive head motion (mean Jenkinson framewise displacement (FD >0.2 mm) or with incomplete imaging data were excluded; (3) individuals in the remission stage, indicated by a Hamilton Depression Rating Scale (HAMD) score of 7 or lower; and (4) subjects lacking either HAMD or Hamilton Anxiety Rating Scale (HAMA) assessments. For more details, refer to Fig. 2. All participants gave written informed consent, and the study was approved by Institutional Review Boards (details in **Supplementary Method**).

The eligible MDD patients were grouped according to their HAMA scores. MDD patients scoring greater than 18 on the HAMA scale were included in the major depressive disorder with significant anxiety (MDD/ANX+) group, whereas those with a HAMA score of 18 or less were included in the major depressive disorder without significant anxiety (MDD/ANX-) group [12].

### 2.2 Data Acquisition, Preprocessing, and FC Analysis

Functional and structural MRI data were collected from nine scanning sites and preprocessed using DPARSF software (http://rfmri.org/DPARSF) following a standardized protocol by the Chinese consortium members [11]. Imaging acquisition parameters are detailed in **Supplementary Table 1**. The preprocessing steps included discarding the initial 10 time points; performing slice-timing correction and realignment; co-registering individual T1-weighted images with the mean functional image; segmenting into gray matter (GM), white matter (WM), and cerebrospinal fluid (CSF); and normalizing. Head motion, WM, and CSF signals, and linear trends, were regressed out. Of those, the Friston 24-parameter model was used to address head motion effects. Temporal bandpass filtering ranging from 0.01 to 0.1 Hz was applied to all time series.

Following preprocessing, we performed the analysis based on two levels: edge-based FCs and network-level FCs. To mitigate significant biases attributable to the varied scanners and sequence parameters used at different sites, we first applied ComBat harmonization to adjust for site effects [13]. The HAMD scores, HAMA scores, age, and sex were included as covariates of interest to be protected. For the edge-based FCs, we extracted time series data from the Dosenbach atlas (160 regions of interest (ROIs)), [14] and these FCs were determined by calculating Fisher’s z-transformed Pearson’s correlation coefficients between each pair of regions, leading to the 160 × 160 FC matrix. In the network-level FC analysis, we categorized all nodes into seven networks as defined in the Yeo atlas, which includes the somatomotor network (SMN), ventral attention network (VAN), visual network (VN), dorsal attention network (DAN), DMN, frontoparietal network (FPN), and subcortical network (SC) [15]. The latter was chosen instead of the limbic network in the Yeo atlas due to the absence of any Dosenbach ROIs within the limbic network. For network-level FC analysis, we averaged the FC values within each network, resulting in a 7 × 7 network matrix.

### 2.3 Brain Gene Expression Data Processing

Gene expression data from the AHBA dataset (http://human.brain-map.org) were acquired, encompassing 20,000 gene expression measures derived from 3702 tissue samples using probes [8]. These tissues were obtained from six human donor brains aged 24 to 57 years, with no recorded neuropsychiatric or neuropathological history.

Subsequently, the abagen toolbox [16] in Python was used to preprocess the gene data and assign samples to the Dosenbach atlas, following these steps: (1) intensity-based filtering was applied to the microarray probes, with those not exceeding 50% background noise being discarded; (2) probes that exhibited a consistent pattern of regional variation analogous to RNAseq data were identified [17]; (3) microarray samples were aligned with the brain parcels defined in the Dosenbach atlas, allowing a maximum distance discrepancy of 10 mm in matching tissue samples to the corresponding atlas regions [6]; (4) gene normalization was performed for all samples, within structural classes, and aggregation was conducted within each brain region, first independently for each donor and then across donors; and (5) only tissue samples from the left hemisphere were included, since all six donors had expression data in the left hemisphere, while only two donors had samples in the right hemisphere [10]. This resulted in gene expression levels of 13,559 genes across 81 regions.

### 2.4 Statistical Analysis

#### 2.4.1 Statistical Analysis of Demographic and Clinical Data

IBM SPSS Statistics Version 26.0 (IBM Corp., Armonk, NY, USA) was used to analyze the demographic and clinical data of the participants. Mann-Whitney U tests were conducted to detect differences in age, years of education, total HAMD-17 scores, and HAMA scores. Differences in sex between the groups were estimated using a Chi-squared test. *p* values < 0.05 were considered statistically significant.

#### 2.4.2 Statistical Analysis of fMRI Data

We initially conducted comparisons of the edge-based and network-level FCs between the MDD/ANX+ and MDD/ANX- groups. These comparisons used a two-sample *t*-test and were corrected using the network-based statistics (NBS) method within DPABI 8.1 (http://rfmri.org/DPABI) [18]. The analysis incorporated covariates including sex, age, head motion (measured by mean FD), and education level. Permutation testing with 1000 iterations was employed, and the results were considered significant at an edge-level *p* value of <0.001 and a component-level *p* value of <0.05, after NBS correction for multiple comparisons.

#### 2.4.3 Statistical Analysis of Gene Expression Data

To assess if brain-wide gene expression from the AHBA atlas can predict FC differences between the MDD/ANX+ and MDD/ANX- groups, we used partial least squares (PLS) regression analysis using the SIMPLS algorithm in MATLAB (plsregress) [6]. Our analysis included 81 brain regions, with 13,559 gene expression values as predictors (x) and the net t-value as the response variable (y), which is calculated as the sum of positive t-values minus the sum of absolute negative t-values for each region [6]. The fitted PLS model can generate several components, and each of them is a linear combination of the predictor variables that can explain variance in the response variables. We kept the component of the PLS model that explained the most variance in the response variables. The significance level of the explained variance of the PLS component was examined via permutation tests based on spatial autocorrelation correction. We further adopted a bootstrapping method to assess and correct for error in estimating the weight of each gene’s contribution to the PLS component. The z-scores of each gene weight in the PLS model were then determined by the ratio of the raw weight to estimated bootstrapping error. Genes were ranked according to their contribution to the PLS component.

### 2.5 Gene Enrichment Analysis

Gene set enrichment analysis was used to explore functional annotations linked to the genes associated with FC discrepancies between the MDD/ANX+ and MDD/ANX- groups. This analysis was carried out using the ToppGene portal (https://toppgene.cchmc.org/) [10]. We selected genes exhibiting PLS weights exceeding a |z|-score of 3 [19] and submitted them to ToppGene to carry out enrichment analyses, including Kyoto Encyclopedia of Genes and Genomes (KEGG) pathways, as well as Gene Ontology (GO) annotations covering molecular functions (MF), biological processes (BP), and cellular components (CC). Additionally, the portal’s disease database was used to identify diseases related to the selected genes. To address multiple comparison concerns, we employed the Benjamini-Hochberg False Discovery Rate (BH-FDR) method, setting the threshold for significant functional annotation at *p* < 0.05 [14].

### 2.6 Validation Analyses

To explore the effects of age and sex on our main results, we conducted validation analyses that involved matching participants by age and sex using propensity score matching. We compared edge-based and network-level FCs between the matched MDD/ANX+ and MDD/ANX- groups using a paired *t*-test. We controlled for the FD and applied NBS correction with 1000 permutations. The results were considered significant at an edge-level *p* value of <0.001 and a component-level *p* value of <0.05, after NBS correction for multiple comparisons.

## 3. Results

### 3.1 Participants

Among the participants with MDD, 294 patients reported HAMA scores greater than 18, forming the MDD/ANX+ group, while 218 patients reported HAMA scores of 18 or less, forming the MDD/ANX- group.

Significant differences were noted in age (*p* < 0.001), sex (*p* = 0.012), and education (*p* = 0.048). The total HAMD-17 scores and HAMA scores in the MDD/ANX+ group were significantly higher than those in the MDD/ANX- group (*p* < 0.001) (Table 1).

**Table 1.** **Demographic and clinical characteristics of the participants included in the analysis**.

Characteristic	MDD/ANX+	MDD/ANX-	*p* value
	(n = 294)	(n = 218)	
Age (y) ^a^	37.5 (28.0, 48.0)	28.0 (21.0, 40.0)	<0.001 *
Sex (% female) ^b^	203 (69.0%)	127 (58.3%)	0.012 *
Education (y) ^a^	11.0 (8.0, 15.0)	12.0 (9.0, 14.0)	0.048 *
FD (mm) ^a^	0.058 (0.042, 0.083)	0.059 (0.043, 0.089)	0.35
Illness duration (months) ^a, c^	24 (5, 60)	24 (6, 60)	0.64
Medication (% using medication) ^a, d^	107 (45.9%)	64 (55.2%)	0.10
First episode (% of first episodes) ^a, e^	205 (76.8%)	97 (63.4%)	0.003 *
HAMD ^a^	23.0 (20.0, 26.0)	19.0 (16.0, 22.0)	<0.001 *
HAMA ^a^	24.0 (21.0, 30.0)	13.0 (9.0, 16.0)	<0.001 *

^a^ Age, education, HAMD, and HAMA scores were observed to have skewed distributions. Therefore, the statistical descriptions use the median and the 25th and 75th percentiles to represent these characteristics. Group comparisons were conducted using the Mann-Whitney U test. ^b^ Group comparisons were performed using the Chi-squared test. ^c^ Illness information is missing for 57 individuals in the MDD/ANX- group and 23 individuals in the MDD/ANX+ group. ^d^ Medication information is missing for 102 individuals in the MDD/ANX- group and 61 individuals in the MDD/ANX+ group. ^e^ First episode information is missing for 65 individuals in the MDD/ANX- group and 27 individuals in the MDD/ANX+ group. * Indicates statistical significance. Note. HAMD, 17-item Hamilton Depression Rating Scale; HAMA, Hamilton Anxiety Rating Scale; MDD/ANX+, MDD patients with significant anxiety; MDD/ANX-, MDD patients without significant anxiety; y, year; FD, Head motion measured by mean Jenkinson framewise displacement.

### 3.2 FC Differences Between the MDD/ANX+ Group and the MDD/ANX- Group

**Fig. 3.**
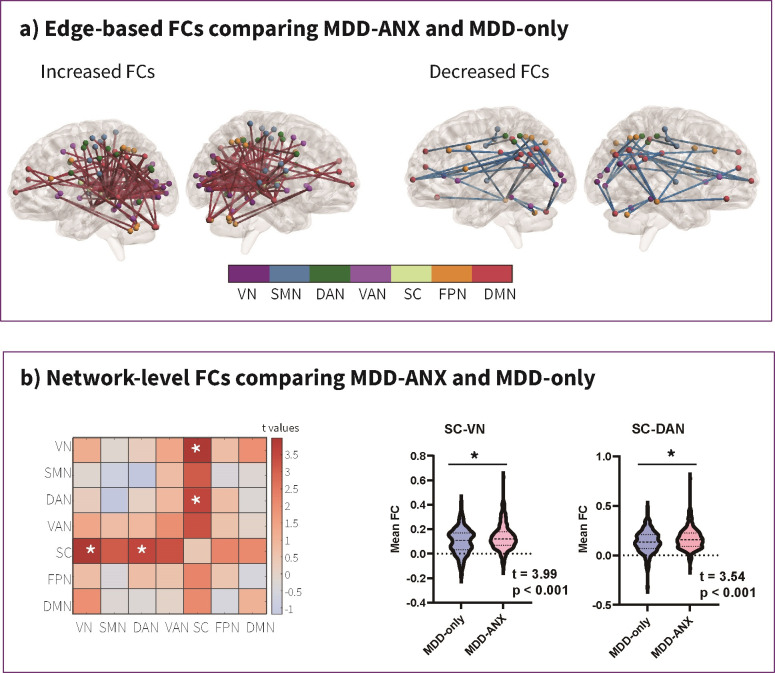
**Functional connectivity disparities between the MDD/ANX+ and MDD/ANX- groups, both at edge-based FCs (a) and network-level FCs (b)**. Note. MDD/ANX+, MDD patients with significant anxiety; MDD/ANX-, MDD patients without significant anxiety; FCs, functional connectivities; SMN, somatomotor network; VAN, ventral attention network; VN, visual network; DAN, dorsal attention network; DMN, default mode network; FPN, frontoparietal network; and SC, subcortical network. * indicates significance.

The MDD/ANX+ patients exhibited significant differences in both edge-based and network-level FCs when compared with the MDD/ANX- group. The significant differences in edge-based FCs and network-level FCs (NBS corrected *p* < 0.05) are illustrated in Fig. 3. For better visualization, we counted and presented the raw number of significant edges falling into each of the within-network and between-network classes in **Supplementary Fig. 1**.

Results from the edge-based and network-level FC analyses were generally consistent with each other, indicating significantly increased FC patterns between the SC and VN, and between the SC and DAN. Specifically, the edge between the SC and VN involves connections between the thalamus/posterior cingulate and occipital areas. Similarly, the edge between the SC and DAN involves connections between the thalamus/posterior cingulate, and the parietal areas.

In addition to the significant FCs between the SC and VN, and the SC and DAN at both edge and network levels, many significant links were observed after NBS correction in the edge FCs analysis, involving the DMN (posterior cingulate, temporal, prefrontal, and occipital cortices; angular gyrus; precuneus; and inferior parietal lobule [IPL]) and FPN (prefrontal cortex, IPL, fusiform gyrus, and inferior cerebellum), as shown in Fig. 3, **Supplementary Fig. 1**, and **Supplementary Table 2**.

### 3.3 Genes Associated with the FC Differences Between the MDD/ANX+ and MDD/ANX- Groups

**Fig. 4.**
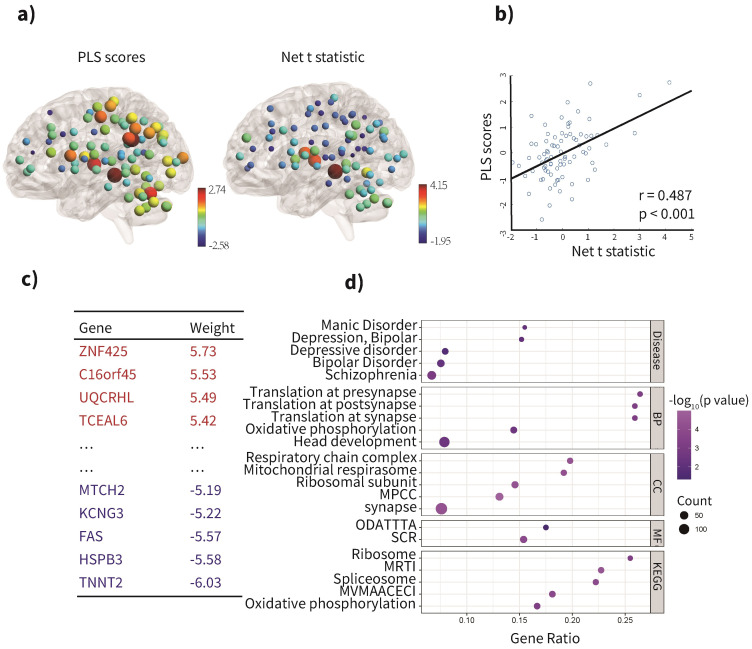
**Spatial correlation between differences in FCs between MDD/ANX+ and MDD/ANX- and gene expression matrix**. (a) Regional PLS scores of genes in the left hemisphere of the Dosenbach Atlas, along with the net t-value representing the FC difference between MDD/ANX+ and MDD/ANX-, were both presented using their z-scores. The color gradient represents the PLS score or net t-value, with positive values displayed in red and negative values in blue, creating a spectrum that reflects the direction and intensity of the scores. The node size corresponds to the absolute value of the PLS score or net t-value, with larger nodes indicating higher absolute values. (b) Scatterplot of PLS scores vs regional t-statistic, basing on z-scores. (c) Genes that positively and negatively weighted PLS values. (d) Gene Ontology (GO), Kyoto Encyclopedia of Genes and Genomes (KEGG), and disease terms for PLS genes. The size of the circle represents the number of genes involved in the specific term, and the color represents the corrected *p* values (*p* < 0.05, FDR-corrected). Note. MDD/ANX+, MDD patients with significant anxiety; MDD/ANX-, MDD patients without significant anxiety; MPCC, Mitochondrial Protein-Containing Complex; ODATTTA, Oxidoreduction-Driven Active Transmembrane Transporter Activity; MRTI, Medicus Reference Translation Initiation; SCR, Structural Constituent of Ribosome; MVMAACECI, Medicus Variant Mutation Caused Aberrant Abeta to Electron Transfer in Complex I; PLS, partial least squares; FCs, functional connectivities.

We next performed transcriptomic analysis to determine which genes were associated with the FC differences between the MDD/ANX+ and MDD/ANX- groups. We found that the third component of PLS (PLS3), accounted for the largest proportion of variance, explaining 23.7% of the variance in FC differences between these groups (Fig. 4a), which was significantly more than expected by chance (*p_permutation* = 0.0110). The spatial distribution of PLS3 positively correlated with the net t-value representing the FC differences between MDD/ANX+ and MDD/ANX- (Fig. 4b, r = 0.487, *p* < 0.001). Of the 1066 genes with a |z|-score >3, 682 were positively weighted and 384 were negatively weighted (Fig. 4c).

### 3.4 Gene Functional Enrichment

To elucidate the molecular functions, biological functions, cellular components, and diseases associated with the genes related to FC differences in MDD/ANX+ patients, we conducted functional enrichment analyses. The PLS3 gene set predominantly associates with biological processes that involve translation at the synapse, pre- and post-synapse, as well as adenosine triphosphate (ATP) production through oxidative phosphorylation. It is associated with cellular components integral to ATP production, such as mitochondrial protein complexes, respiratory chain complexes, and the mitochondrial respirasome, in addition to components related to the synapse and translation processes (e.g., ribosomal subunits). On a molecular level, its functions are crucial for translation (acting as a structural component of the ribosome) and for ATP generation (through oxidoreduction activities). Furthermore, this gene set is related to KEGG pathways concerning translation processes (ribosome, mediating factors for translation initiation, and the spliceosome) as well as ATP generation (a KEGG MEDICUS variant mutation causes aberrant abeta to electron transfer in complex I and oxidative phosphorylation). Notably, there is a significant correlation between the PLS3 gene set and mood disorders, such as depressive disorders and bipolar disorder, encompassing both manic and depressive phases, as illustrated in Fig. 4d.

### 3.5 Validation Analyses

Overall, the demographic data of the matched groups showed no significant differences (*p* > 0.05) in age, sex, and education (**Supplementary Table 3**). The FC differences observed were consistent across both the main findings and the validation analysis (r = 0.931, *p* < 0.001), where participants were matched based on age and sex (**Supplementary Fig. 2**). The results from the validation analysis were similar to those of the main analysis, affirming the reliability of our main findings when accounting for age and sex influences (**Supplementary Fig. 2**).

## 4. Discussion

To our knowledge, this is the first study to investigate gene expression related to FC differences between MDD/ANX+ and MDD/ANX-. Results from both edge-based and network-level FC analyses were generally consistent with each other, indicating significantly increased FC patterns in the SC and VN, as well as between the SC and DAN, in patients with MDD/ANX+ compared with those with MDD/ANX-. Additionally, the transcriptome-neuroimaging correlation analysis revealed that the expression of 1066 genes was spatially correlated with the FC differences between MDD/ANX+ and MDD/ANX-. These genes demonstrated enrichment in crucial molecular functions, biological processes, and cellular components related to translation at synapses and ATP generation. They were also associated with some common mental disorders, including depressive disorders and bipolar disorder. This study may provide insights into the intricate interplay between gene expression and FC differences in MDD/ANX+.

Our study has identified significantly increased FC patterns within the SC and VN, as well as between the SC and DAN, in patients with MDD/ANX+ compared with those with MDD/ANX-. This observation aligns with the existing scientific literature on somatic anxiety. For example, patients with somatization disorder have shown enhanced connectivity between the thalamus and the visual cortex [20]. Similarly, research has found impaired intrinsic FC between the thalamus and visual cortex in individuals with migraine without aura [21]. The increased connectivity observed in our MDD/ANX+ group, especially involving the SC, VN, and DAN, may reflect a neural mechanism underlying the heightened alertness, internal focus, and altered visual function characteristic of anxiety co-occurring with depression [22].

Leveraging transcription-neuroimaging spatial association analysis, 1066 genes were found to be spatially correlated with the FC differences between MDD/ANX+ and MDD/ANX-. These genes demonstrated enrichment in crucial molecular functions, biological processes, and cellular components related to translation at synapses (ribosome; mediating factors for translation initiation; spliceosome; and translation at the synapse, pre- and post-synapse) and ATP generation (a KEGG MEDICUS variant mutation causes aberrant abeta to electron transfer in complex I, oxidative phosphorylation, mitochondrial protein complexes, respiratory chain complexes, and the mitochondrial respirasome). Depression and anxiety are widely understood to stem from maladaptive alterations in particular brain regions and circuits, with synaptic changes playing a crucial role. Research has shown that depression and anxiety are linked to a reduction in neuronal synapses within key brain regions governing mood and cognition, such as the prefrontal cortex [23]. Furthermore, treatments for depression and anxiety, such as ketamine, have been observed to swiftly promote synaptogenesis, effectively countering the synaptic deficits induced by prolonged stress [24].

The recent proposition of a mitochondrial etiology for neuropsychiatric disorders underscores the critical role of ATP in the functioning of the brain [25]. Given the high energy demands of neurons, their reliance on ATP—produced primarily by mitochondria and supported by astrocytes—is paramount [26]. ATP, known as the biological energy currency, drives enzyme activity across all cells and tissues, extending beyond mere energy storage to function as an excitatory neurotransmitter or neuromodulator [27]. Emerging research suggests that inadequate ATP release from astrocytes may contribute to the onset of depression and anxiety [28, 29] and the exogenous supplementation of extracellular ATP, or the stimulation of endogenous ATP release from astrocytes, shows promise in alleviating these conditions [30]. In terms of disease, the enrichment of genes associated with comorbid anxiety in MDD is observed in several mental disorders, including depression, bipolar disorder, and schizophrenia, indicating some common genetic mechanisms underlying these conditions. Currently, measuring regional gene expression in the brain *in vivo* is extremely difficult. Thus, our results offer a preliminary clue in explaining the relationship between these microscale biological events and macroscopic network in MDD.

Several limitations should be acknowledged. First, the AHBA gene expression data were collected from healthy participants, while our neuroimaging data were derived from individuals with MDD. Therefore, the gene expression findings in our study need further validation using a large sample of whole-brain gene expression data from MDD patients. Second, given the limited right hemisphere gene expression data, we only included tissue samples from the left hemisphere gene expression data in the study, which may result in some bias. Third, all our study subjects are from China and may not fully reflect the differences in gene expression between different populations. Fourth, we used the REST-meta-MDD dataset, which includes multi-site data; site effects may have still influenced the results even though we applied the ComBat method for correction. Future studies should consider validation using single-site data with consistent scanning parameters. Fifth, we only used ComBat for site correction, and we recommend that future research explore alternative or more advanced harmonization techniques to further validate our findings. Sixth, we used the REST-meta-MDD database, which only provides data on age, sex, education level, HAMD, HAMA, illness status, medication status, and whether it was a first episode. We suggest that future studies incorporate more extensive demographic and clinical data to better assess their influence on FC.

## 5. Conclusions

To our knowledge, this is the first study to investigate gene expression related to FC differences between MDD/ANX+ and MDD/ANX-. Our findings reveal significantly increased FC patterns between the SC and VN, as well as between the SC and DAN in MDD/ANX+ patients compared with MDD/ANX- patients. These differences may be modulated by the differential expression of specific genes related to synaptic translation and ATP generation. The expression differences may increase the risk for MDD with anxiety symptoms and may be associated with poorer functional status and treatment outcomes.

## Availability of Data and Materials

The data from the REST-meta-MDD project can be accessed at http://rfmri.org/REST-meta-MDD. Gene expression data is publicly available through the Allen Human Brain Atlas dataset at http://human.brain-map.org/static/download.

## Supplementary Material

Supplementary material associated with this article can be found, in the online version, at https://doi.org/10.31083/AP39865.
