# Supplementary material for: Regional Gene Expression Patterns are Associated with Functional Connectivity Alterations in Major Depressive Disorder with Anxiety Symptoms
Source: Alpha Psychiatry. 2025 Apr 21;26(2):39865. doi: 10.31083/AP39865 (PMC12059726; doi:10.31083/AP39865)
Supplement: Supplementary file 1 [file 2757-8038-26-2-39865-s1.doc]

***Regional Gene Expression Patterns Are Associated with Functional Connectivity Alterations in Major Depressive Disorder with Anxiety Symptoms***

*Supplemental Information*

**Summary**

**Supplementary methods**

**Supplementary Figure S1.** The network distribution of the significant edge from the results of the edge-based FCs analysis.

**Supplementary Figure S2.** Results of the validation analysis.

**Supplementary Table S1.** fMRI data acquisition parameters and sample size for each study site included.

**Supplementary Table S2.** The results of the edge-based FC comparison between MDD/ANX+ and MDD/ANX-.

**Supplementary Table S3.** Demographic and clinical characteristics of the participants included in the validation analysis.

**Supplementary methods**

***Participants***

This study was based on the REST-meta-MDD Project, which provided fMRI and clinical data from 1300 patients diagnosed with Major Depressive Disorder (MDD) [1]. These patients were recruited from 25 Chinese consortium members across 17 hospitals at their respective sites [1]. All patients provided written informed consent, and the original studies received approval from local Institutional Review Boards before participation in the study. Subsequently, the sharing of deidentified and anonymized data was approved by the Institutional Review Board of the Institute of Psychology, Chinese Academy of Sciences [1]. Depression was measured using the Hamilton Depression Rating Scale (HAMD-17), and anxiety was measured using the Hamilton Anxiety Rating Scale (HAMA).

We selected 512 MDD patients for analysis based on the following criteria: 1) Site 4 was excluded due to duplication from site 14, resulting in 1226 MDD. 2) Site 25 was excluded as it mainly contained late-onset depression (mostly with age > 60) and remitted patients, resulting in 1137 MDD. 3) Subjects with poor imaging data (quality control scores < 4) and incomplete imaging data were excluded, resulting in 1048 MDD. 4) Those in the remission stage (HAMD score ≤ 7) were excluded, resulting in 1015 MDD. 5) Individuals with excessive head motion (mean Jenkinson framewise displacement > 0.2 mm) were excluded, resulting in 997 MDD. 6) Those with both HAMD and HAMA measurements were included, resulting in 512 MDD.


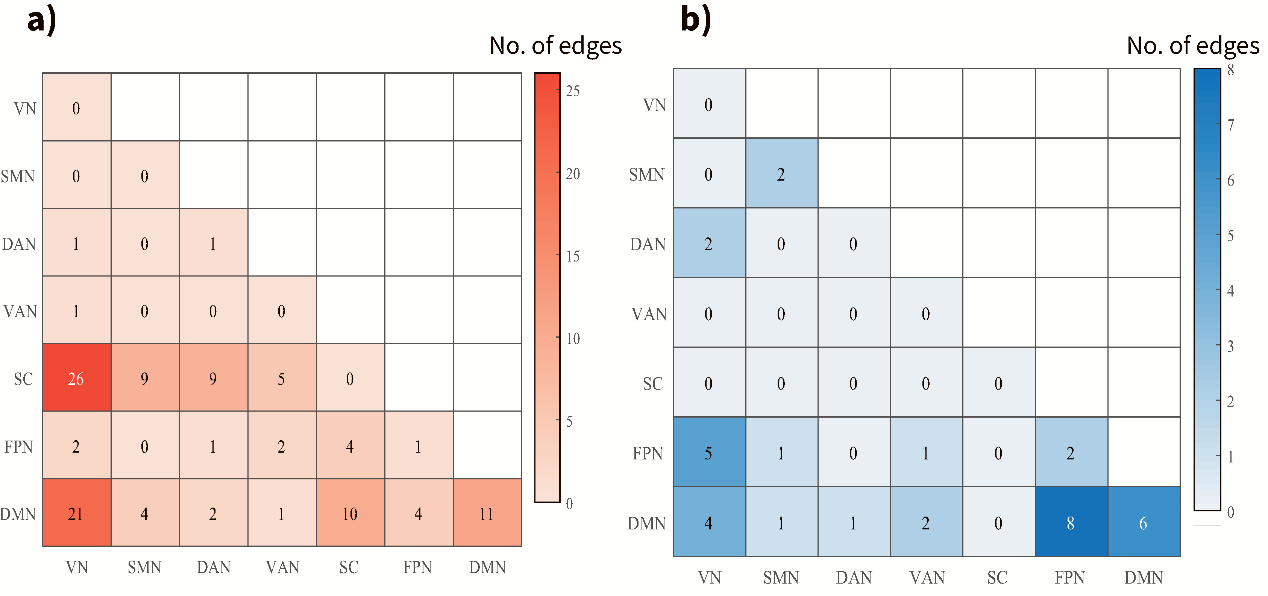


**Supplementary Figure S1.** The network distribution of the significant edge from the results of the edge-based FCs analysis.

*Note. FCs, functional connectivities; SMN, Somatomotor Network; VAN, Ventral Attention Network; VN, Visual Network; DAN, Dorsal Attention Network; DMN, Default Mode Network; FPN, Frontoparietal Network; and SC, Subcortical Network.*


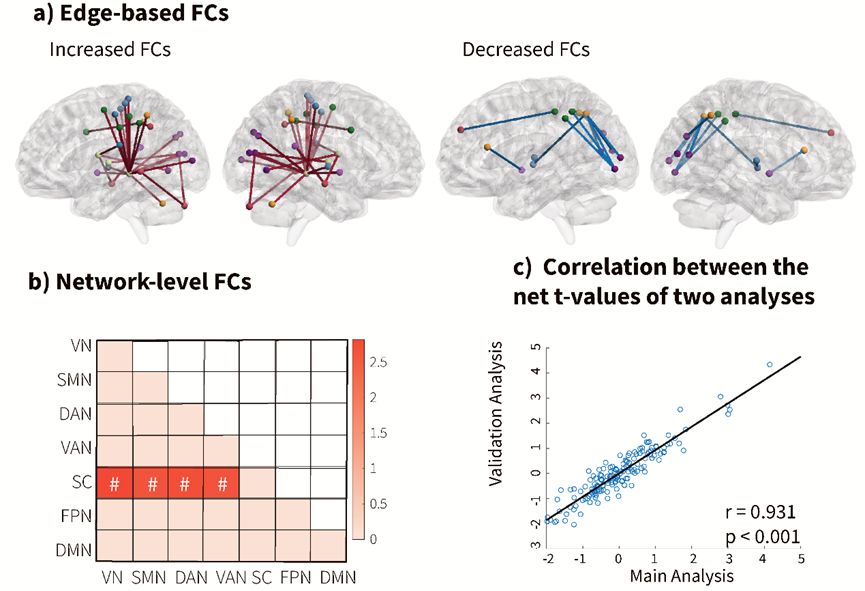


**Supplementary Figure S2.** Results of the validation analysis.

*Note. MDD/ANX+, MDD patients with significant anxiety; MDD/ANX-, MDD patients without significant anxiety; FCs, functional connectivities; SMN, somatomotor network; VAN, ventral attention network; VN, visual network; DAN, dorsal attention network; DMN, default mode network; FPN, frontoparietal network; and SC, subcortical network. "#" indicates significance at the level of an uncorrected p < 0.01.*

**Supplementary Table S1. fMRI Data Acquisition Parameters and Sample Size for Each Study Site Included.**

| Cohort number | Sample size | Scanner | Receive (coil) | TR (ms) | TE (ms) | Flip Angle (︒) | Thickness/gap | Slice number | Time points | Voxel size | FOV |
| --- | --- | --- | --- | --- | --- | --- | --- | --- | --- | --- | --- |
| 7 | 38 | GE discovery MR750 | 8 channel | 2000 | 30 | 90 | 3.2mm/0mm | 37 | 184 | 2.29 × 2.29 × 3.20 | 220 × 220 |
| 8 | 55 | GE Signa 3T | 8 channel | 2000 | 30 | 90 | 3.0mm/0mm | 35 | 200 | 3.75 × 3.75 × 3.00 | 240 × 240 |
| 10 | 30 | Siemens Tim Trio 3T | 32 channel | 2000 | 30 | 90 | 3.0mm/1.52mm | 32 | 212 | 3.75 × 3.75 × 4.52 | 240 × 240 |
| 17 | 47 | GE Signa 3T | 8 channel | 2000 | 40 | 90 | 4.0mm/0mm | 33 | 240 | 3.75 × 3.75 × 4.00 | 240 × 240 |
| 18 | 12 | Philips Achieva 3.0 T scanner | 8 channel | 2000 | 35 | 90 | 5.0mm/1.0 mm | 24 | 200 | 1.67 × 1.67 × 6.00 | 240 × 240 |
| 20 | 240 | Siemens Tim Trio 3T | 12 channel | 2000 | 30 | 90 | 3.0mm/1.0mm | 32 | 242 | 3.44 × 3.44 × 4.00 | 220 × 220 |
| 21 | 59 | Siemens Tim Trio 3T | 32 channel | 2000 | 30 | 90 | 3.5mm/0.7mm | 33 | 240 | 3.12 × 3.12 × 4.20 | 200 × 200 |
| 22 | 25 | Philips Gyroscan Achieva 3.0T | 32 channel | 2000 | 30 | 90 | 4.0mm/0mm | 36 | 250 | 1.67 × 1.67 × 4.00 | 240 × 240 |
| 24 | 6 | GE Signa 1.5T | 8 channel | 2000 | 40 | 90 | 5mm/1mm | 24 | 160 | 3.75 × 3.75 × 6.00 | 240 × 240 |

*Note. FOV, field of vision; TE, time of echo; TR, time of repetition.*

**Supplementary Table S2. The results of the edge-based FC comparison between MDD/ANX+ and MDD/ANX-.**

| ROI1 | ROI2 | t value | p value |
| --- | --- | --- | --- |
| vmPFC | post cingulate | -3.419727877 | 0.000677475 |
| vmPFC | IPL | 3.367897023 | 0.000815153 |
| vmPFC | angular gyrus | 3.63788836 | 0.000303082 |
| vmPFC | post cingulate | -4.125833456 | 4.32E-05 |
| vmPFC | occipital | -3.509877926 | 0.00048834 |
| mPFC | post parietal | -4.485853526 | 9.00E-06 |
| aPFC | ant insula | 3.511574649 | 0.000485307 |
| aPFC | thalamus | 3.537460114 | 0.000441173 |
| vmPFC | fusiform | -3.715998947 | 0.000224964 |
| vmPFC | occipital | 3.404766913 | 0.000714809 |
| vlPFC | fusiform | -3.399046787 | 0.000729582 |
| vPFC | angular gyrus | -3.622270521 | 0.00032149 |
| vPFC | post cingulate | 3.608610969 | 0.000338444 |
| dlPFC | occipital | -4.169365296 | 3.59E-05 |
| ant insula | inf cerebellum | 3.324982116 | 0.000948404 |
| ant insula | inf cerebellum | 3.336483228 | 0.000910835 |
| ant insula | angular gyrus | -3.620912948 | 0.000323138 |
| mFC | occipital | 3.425622913 | 0.00066327 |
| vFC | angular gyrus | -3.382964326 | 0.000772659 |
| dFC | IPL | 3.425575903 | 0.000663383 |
| dFC | inf cerebellum | 3.336162221 | 0.000911865 |
| dFC | fusiform | -3.44452414 | 0.000619577 |
| dFC | inf cerebellum | 3.364980735 | 0.000823625 |
| vFC | basal ganglia | 4.509580435 | 8.08E-06 |
| pre-SMA | thalamus | 3.404802768 | 0.000714717 |
| mid insula | thalamus | 3.361627592 | 0.000833468 |
| mid insula | post cingulate | 4.198851061 | 3.17E-05 |
| precentral gyrus | post cingulate | 4.075927498 | 5.32E-05 |
| thalamus | inf temporal | 3.875520915 | 0.000120396 |
| thalamus | lat cerebellum | 3.540448765 | 0.000436327 |
| thalamus | occipital | 3.86735363 | 0.000124378 |
| thalamus | occipital | 3.639093158 | 0.000301704 |
| thalamus | post occipital | 3.424807828 | 0.000665218 |
| thalamus | post occipital | 3.42948962 | 0.000654104 |
| precentral gyrus | thalamus | 3.883185032 | 0.000116769 |
| parietal | post cingulate | 3.848906143 | 0.000133836 |
| precentral gyrus | post cingulate | 3.41761277 | 0.00068264 |
| precentral gyrus | parietal | -3.56655219 | 0.000396067 |
| precentral gyrus | precuneus | -3.405845018 | 0.000712055 |
| parietal | sup temporal | 3.691109043 | 0.000247521 |
| thalamus | inf temporal | 3.377887185 | 0.000786742 |
| thalamus | IPL | 3.459772497 | 0.000586301 |
| thalamus | sup temporal | 3.377556187 | 0.000787668 |
| thalamus | lat cerebellum | 3.925819509 | 9.84E-05 |
| thalamus | occipital | 3.364985129 | 0.000823613 |
| thalamus | occipital | 4.403756679 | 1.30E-05 |
| thalamus | post occipital | 3.714078866 | 0.000226633 |
| thalamus | post occipital | 3.764326317 | 0.000186583 |
| thalamus | post occipital | 3.386088466 | 0.00076411 |
| thalamus | post occipital | 3.434880433 | 0.000641523 |
| thalamus | sup temporal | 3.679182008 | 0.000259068 |
| thalamus | occipital | 5.208887168 | 2.77E-07 |
| thalamus | occipital | 3.561371519 | 0.00040377 |
| thalamus | post occipital | 3.674469711 | 0.000263769 |
| thalamus | post occipital | 3.704301021 | 0.000235313 |
| thalamus | post occipital | 3.529314776 | 0.000454638 |
| thalamus | post occipital | 3.523456109 | 0.00046456 |
| mid insula | post cingulate | 3.510266594 | 0.000487643 |
| mid insula | IPS | -3.386251055 | 0.000763667 |
| parietal | angular gyrus | 3.67801532 | 0.000260225 |
| inf temporal | basal ganglia | 3.358149806 | 0.000843792 |
| inf temporal | post cingulate | 4.453058251 | 1.04E-05 |
| inf temporal | sup temporal | 3.899451382 | 0.00010941 |
| inf temporal | occipital | 3.670459088 | 0.000267832 |
| parietal | post cingulate | 3.860364614 | 0.000127884 |
| parietal | parietal | -3.39112046 | 0.000750525 |
| parietal | post cingulate | 4.710366685 | 3.20E-06 |
| precentral gyrus | post cingulate | 4.159777657 | 3.74E-05 |
| parietal | post cingulate | 4.448029268 | 1.07E-05 |
| parietal | angular gyrus | 3.407887257 | 0.000706867 |
| post insula | occipital | 3.578898629 | 0.000378263 |
| basal ganglia | sup temporal | 3.912942824 | 0.000103642 |
| basal ganglia | occipital | 3.490683195 | 0.000523903 |
| post cingulate | precuneus | -3.321605556 | 0.000959704 |
| post cingulate | post cingulate | -3.346990298 | 0.000877728 |
| parietal | post cingulate | 3.810863145 | 0.000155529 |
| temporal | post cingulate | 3.633152424 | 0.000308557 |
| post parietal | post cingulate | 4.045353995 | 6.04E-05 |
| post cingulate | sup parietal | 3.335588874 | 0.000913706 |
| post cingulate | IPL | 4.077601116 | 5.28E-05 |
| post cingulate | inf temporal | 4.361498073 | 1.57E-05 |
| post cingulate | post parietal | 4.129063549 | 4.26E-05 |
| post cingulate | sup temporal | 3.607075735 | 0.000340401 |
| post cingulate | IPL | 3.457084643 | 0.000592043 |
| post cingulate | IPL | 3.408558301 | 0.00070517 |
| post cingulate | IPS | 3.399464511 | 0.000728494 |
| post cingulate | inf cerebellum | 3.824383912 | 0.000147464 |
| post cingulate | occipital | 3.310440902 | 0.000997964 |
| post cingulate | occipital | 3.766334647 | 0.00018513 |
| post cingulate | occipital | 3.560080414 | 0.000405711 |
| post cingulate | occipital | 4.707742825 | 3.24E-06 |
| post cingulate | occipital | 3.450931524 | 0.000605385 |
| post cingulate | post occipital | 3.671139591 | 0.000267139 |
| post cingulate | post occipital | 4.158494759 | 3.76E-05 |
| post cingulate | post occipital | 3.64173166 | 0.000298707 |
| post cingulate | post occipital | 3.585335713 | 0.000369281 |
| post cingulate | post occipital | 3.872747049 | 0.000121734 |
| fusiform | post cingulate | -3.872436287 | 0.000121885 |
| fusiform | precuneus | -3.338219318 | 0.000905286 |
| fusiform | angular gyrus | -3.414163749 | 0.000691142 |
| fusiform | occipital | -3.572284378 | 0.000387706 |
| fusiform | post cingulate | 3.409485859 | 0.000702831 |
| fusiform | post cingulate | 3.689616607 | 0.000248939 |
| fusiform | inf cerebellum | -3.383640446 | 0.000770801 |
| fusiform | inf cerebellum | -3.486038271 | 0.000532866 |
| precuneus | sup temporal | 3.953082838 | 8.81E-05 |
| precuneus | IPS | 3.724994824 | 0.000217299 |
| IPL | IPL | 3.839388941 | 0.000138978 |
| IPL | post occipital | -3.467459126 | 0.000570167 |
| post cingulate | IPS | 3.345954034 | 0.000880942 |
| post cingulate | post occipital | 3.3741668 | 0.000797213 |
| inf temporal | sup temporal | 3.316935613 | 0.000975538 |
| inf temporal | inf cerebellum | 3.528303965 | 0.000456336 |
| inf temporal | inf cerebellum | 3.741753562 | 0.00020367 |
| occipital | post cingulate | 3.415178076 | 0.000688632 |
| occipital | post cingulate | 4.325313451 | 1.84E-05 |
| occipital | IPS | 3.494606024 | 0.000516444 |
| occipital | occipital | 3.78736344 | 0.000170544 |
| post cingulate | post cingulate | -4.03004351 | 6.43E-05 |
| IPL | occipital | 3.671558261 | 0.000266713 |
| post parietal | post occipital | -3.487245898 | 0.000530522 |
| sup temporal | post occipital | 3.573385898 | 0.000386118 |
| sup temporal | post occipital | 3.331927377 | 0.000925549 |
| IPL | inf cerebellum | 3.542733804 | 0.000432655 |
| angular gyrus | post cingulate | 3.463947664 | 0.000577485 |
| angular gyrus | precuneus | 3.744682222 | 0.000201373 |
| angular gyrus | inf cerebellum | 3.521211836 | 0.000468414 |
| angular gyrus | occipital | 3.422603048 | 0.000670512 |
| angular gyrus | post occipital | 4.082039554 | 5.19E-05 |
| angular gyrus | post occipital | 3.844656586 | 0.000136109 |
| angular gyrus | post occipital | 3.823285877 | 0.000148104 |
| IPL | occipital | 3.730111628 | 0.000213049 |
| IPL | post occipital | 3.935664459 | 9.46E-05 |
| IPL | post occipital | 3.716186754 | 0.000224802 |
| IPL | post occipital | 3.541438006 | 0.000434734 |
| IPL | post occipital | 3.860420988 | 0.000127856 |
| IPL | occipital | -3.754534742 | 0.000193822 |
| inf cerebellum | occipital | 3.432738816 | 0.000646494 |
| precuneus | occipital | -3.487068963 | 0.000530864 |
| lat cerebellum | inf cerebellum | -3.42715196 | 0.000659632 |
| IPS | post occipital | -4.18616564 | 3.35E-05 |
| IPS | post occipital | -4.230433079 | 2.77E-05 |
| IPS | post occipital | -3.445620791 | 0.000617126 |
| post cingulate | IPS | 3.421968085 | 0.000672044 |
| occipital | occipital | 3.494123467 | 0.000517356 |
| angular gyrus | occipital | -3.599186991 | 0.000350626 |
| occipital | occipital | 3.431519377 | 0.00064934 |
| inf cerebellum | post occipital | -3.535213015 | 0.00044485 |
| inf cerebellum | post occipital | -3.533938721 | 0.000446948 |
| occipital | inf cerebellum | 3.622256852 | 0.000321506 |

*Note. vmPFC, ventromedial prefrontal cortex; mPFC, medial prefrontal cortex; aPFC, anterior prefrontal cortex; vlPFC, ventrolateral prefrontal cortex; vPFC, ventral prefrontal cortex; dlPFC, dorsolateral prefrontal cortex; mFC, medial frontal cortex; dFC, dorsal frontal cortex; vFC, ventral frontal cortex; pre-SMA, pre-supplementary motor area; IPL, inferior parietal lobule; IPS, intra-parietal sulcus; ant insula, anterior insula; mid insula, middle insula; inf temporal, inferior temporal; sup temporal, superior temporal; lat cerebellum, lateral cerebellum; inf cerebellum, inferior cerebellum; sup parietal, superior parietal; post occipital, posterior occipital; post cingulate, posterior cingulate; post parietal, posterior parietal.*

**Supplementary Table S3.** Demographic and clinical characteristics of the participants included in the validation analysis.

| Characteristics | MDD/ANX+ (n = 166) | MDD/ANX- (n = 166) | p |
| --- | --- | --- | --- |
| Age (y) ^a^ | 32.0 (24.0, 41.0) | 31.0 (23.0, 42.0) | 0.917 |
| Sex (% Female) ^b^ | 105 (63.3%) | 104 (62.7%) | 0.910 |
| Education (y) ^a^ | 9.0 (11.0, 15.0) | 11.0 (8.0, 13.0) | 0.711 |
| HAMD ^a^ | 23.0 (19.0, 25.0) | 19.0 (16.0, 22.0) | < .001* |
| HAMA ^a^ | 24.0 (21.0, 29.0) | 13.0 (10.0, 16.0) | < .001* |

*^a^ Age, education, HAMD, and HAMA scores were observed to have skewed distributions. Therefore, the statistical descriptions utilize the median and the 25th and 75th percentiles to represent these characteristics. Group comparisons were conducted using the Mann-Whitney U test;*

*^b^ Group comparisons were performed using the Chi-square test;*

** Indicates statistical significance.*

Reference

[1] Yan CG, Chen X, Li L, Castellanos FX, Bai TJ, Bo QJ, et al. Reduced default mode network functional connectivity in patients with recurrent major depressive disorder. Proceedings of the National Academy of Sciences of the United States of America. 2019; 116: 9078–9083. https://doi.org/10.1073/pnas.1900390116.
